# Determination of the mean aerosol residence times in the atmosphere and additional ^210^Po input on the base of simultaneous determination of ^7^Be, ^22^Na, ^210^Pb, ^210^Bi and ^210^Po in urban air

**DOI:** 10.1007/s10967-012-1690-5

**Published:** 2012-02-29

**Authors:** Magdalena Długosz-Lisiecka, Henryk Bem

**Affiliations:** Institute of Applied Radiation Chemistry, Technical University of Lodz, Zeromskiego Street 116, 90-924 Lodz, Poland

**Keywords:** Radionuclides, Aerosols, Residence time

## Abstract

The significant differences in the activities of ^210^Pb, ^210^Bi, ^210^Po and cosmogenic ^7^Be and ^22^Na radionuclides in the urban aerosol samples collected in the summers 2010 and 2011 in the Lodz city of Poland were observed. Simultaneous measurement of these radionuclides, after a simple modification of the one compartment model, allows us to calculate both: the corrected aerosol residence times in the troposphere (1 ÷ 25 days) and in the lower stratosphere (103 ÷ 205 days). The relative input of the additional sources (beside of the ^222^Rn decay in the air) to the total activity concentrations of ^210^Pb, ^210^Bi and ^210^Po radionuclides in the urban air, plays a substantial role (up to 97% of the total activity) only in the case of ^210^Po.

## Introduction

Gaseous ^222^Rn escaping from the soil produces in the lower atmosphere a series of longer lived products: ^210^Pb (22.3 years), ^210^Bi (5 days) and ^210^Po (138 days). Since atmospheric aerosols can be transported over long distances, ^210^Pb and its progeny concentrations in a particular site may not be connected strictly with the radium activity in adjacent soils but also on meteorological conditions such as: temperature, wet deposition (rainfall) and wind intensity. These radionuclides are readily absorbed on the surface of aerosol particles, and whereas the activity of ^210^Pb does not change too much, the activities of ^210^Bi and ^210^Po grow during the residence time of aerosol particles in the air. Therefore, the activity ratios of ^210^Bi/^210^Pb and ^210^Po/^210^Pb can be useful for tracing the fate of an aerosol and the estimation of its solid particles’ lifetimes (residence time) [1].

The simple solution of the ^210^Pb, ^210^Bi and ^210^Po activities relationships assume that the only source of the ^210^Bi and ^210^Po in the lower atmosphere (troposphere) is decay ^210^Pb from the ^222^Rn escaping from soil [2]. Commonly used formulas for the aerosol residence time calculation are based on measurement of the isotope ratios of ^210^Po/^210^Pb and ^210^Bi/^210^Pb and application of the steady state equilibrium in one compartment model [3–5]. In this model, the decay of ^222^Rn nuclide in the air is recognized as an only source of airborne ^210^Pb and consequently its daughters: ^210^Bi and ^210^Po (the air is considered as well-mixed reservoir). This assumption does not take into account the possibility of additional emission of these radionuclides from other sources such as re-suspension of soil in summer, the combustion of coal in the winter, incursions of stratospheric air or emission from anthropogenic sources [6].

However, in general, the residence times determined from the ^210^Po/^210^Pb ratios (2–240 days) are usually much longer of those determined from ^210^Bi/^210^Pb ratios (2–25 days) [7]. It indicates that some additional inputs of ^210^Po into troposphere must exist. The evidence of the complementary ^210^Po source from the lower stratosphere input (after volcanic eruption) [8] or soil dust [9] as well as from anthropogenic activities (coal combustion) was also recently documented [10–13]. If these additional ^210^Po sources are taken into account, from comparison of two activities ratio: ^210^Bi/^210^Pb and ^210^Po/^210^Pb the so called corrected residence time *T*_RC_ can be calculated [9].

Very recently [14] published the mathematical solutions for true dependence of ^210^Po/^210^Pb and ^210^Bi/^210^Pb aerosol particles with the additional non-equilibrated ^210^Bi and ^210^Po radionuclide inputs. The problem ^210^Po sources and its ratio to the parent nuclide in the atmosphere is more complex, since after volcanic eruption significant amounts of ^210^Po activities also ejected to the stratosphere [8]. The residence time of the solid particles in the stratosphere is usually remarkably longer than those in the lower layers of troposphere where they are finally transported [15]. Moreover, after interactions of the cosmic radiation with nitrogen and oxygen molecules (in the upper troposphere border) two cosmogenic radionuclides: ^7^Be and ^22^Na, are produced. These radionuclides together with ^210^Pb, ^210^Bi and ^210^Po can be also attached to solid particles moving from stratosphere to troposphere. Therefore, ^7^Be and ^22^Na can also serve as the additional markers to assess the flow of dust into the lower atmosphere [5].

The instrumental γ ray-spectrometry of the collected dust allows beside of ^210^Pb on simultaneous determination of ^7^Be and ^22^Na in the aerosol samples. After radiochemical separation and measurement of ^210^Bi and ^210^Po in these samples, it would be possible to calculate not only the corrected residence time *T*_RC_ of aerosol from ^210^Pb, ^210^Bi and ^210^Po ratios in the surface air, but also on the basis of ^7^Be to ^22^Na ratio, one can calculate the resident times of the aerosol in the lower stratosphere *T*_S_ [16, 17].

Therefore, it seems to be interesting to use modern radionuclide methods (α and γ spectrometry as well as liquid scintillation) for simultaneous determination of ^7^Be, ^22^Na, ^210^Pb, ^210^Bi, ^210^Po in the same aerosol samples in order to trace their fate from the stratosphere to the surface layer of urban air.

## Materials and methods

The air particulate matter samples were collected in the centre of the Polish city of Lodz, by the ASS-500 station operating as part of the national network for monitoring radiation in the air [18]. Approximately 50,000 m^3^ of air was filtered and 2.5–6 g of dust was captured by the quadratic (40 cm × 40 cm) Petrianov type filters during a typical one-week collection period. For further radioactivity measurements, the filters were divided into two equal parts. From the first part, used for fast radiochemical separation of ^210^Bi and ^210^Po, three small discs with a diameter of 5 cm were cut off (5.7% of the total dust) for ^210^Po determination by α spectrometry after its spontaneous deposition on silver plates by a procedure described by us elsewhere [12].

The ^210^Bi was immediately eluted by 100 mL of 2 M HCl solution from the remaining first part of the filter. The elution solution was diluted with water to volume of 200 mL and ^210^Bi radionuclide was concentrated by column chromatography with DOWEX 1 × 8 resin. ^210^Pb and ^210^Po radionuclides were eluted from the column by washing with 50 mL of water followed by passing of 50 mL of 0.1 M HNO_3_. ^210^Bi radionuclide was eluted by 100 mL of 1.8 M H_2_SO_4_, and was extracted directly to the liquid scintillation vials, form this solution with two 5 mL portions of 5% (w/v) trioctylphospine oxide in toluene. Finally before the liquid scintillation counting of ^210^Bi 10 mL of Ultima Gold F cocktail was added. No additional activities of ^210^Pb or ^210^Po were observed in the liquid scintillation spectrum.

The average recovery of ^210^Bi from the filters by this method was determined by comparison of the ^210^Pb and ^210^Bi activities for the old filters, with ^210^Pb–^210^Bi in a radioactive equilibrium state. The average recovery of ^210^Bi was equal to 0.8 ± 0.1.

The homogeneity of the ^210^Po distribution in the filter sheet was checking by comparison of its activity in the 9 small discs taken from the different parts of the sheet. The ^210^Po activity dispersion not exceeded ±3% from the an average value. Therefore, one can assume that activity of ^210^Po is uniformly distributed over the whole filter.

The second part of the filter, after overnight drying, was pressed into the standard disc geometry: 0.4 cm thick, with a diameter of 5.2 cm, for instrumental γ-spectrometry determinations of ^7^Be, ^22^Na and ^210^Pb. The determination limits (with 10% accuracy) according Curie’s formula [19] were for these radionuclides: 3, 0.3 and 1.1 μBq/m^3^, respectively.

### Quality assurance

The accuracy of the used analytical procedures and instrumental γ-spectrometry measurements was checked using the following standard reference materials: IAEA Soil 327, IAEA Sediment 300, Soil Cu-2006-3. A good compliance of the determined values with those certified has been achieved, as previously reported [12, 20].

## Result and discussion

In order to evaluate the possible additional input of ^210^Pb, ^210^Bi and ^210^Po activities—Δ*A*_i_ from both anthropogenic (coal combustion) as well as from natural sources (stratospheric inflow and soil resuspension), we assumed the existence of radioactive equilibrium between these radionuclides introduced additionally to surface air:

1 $$ \Updelta A_{\text{Pb}} = \Updelta A_{\text{Bi}} \; = \;\Updelta A_{\text{Po}} $$

The total observed (measured) activities—*A*_ti_ of these three radionuclides in the air will be the sum of the activities coming from the escaping ^222^Rn decay—*A*_Rni_ and the additional input:

2 $$ A_{\text{mPb}} \; = \;A_{\text{RnPb}} \; + \;\Updelta A_{\text{Pb}} $$

3 $$ A_{\text{mBi}} \; = \;A_{\text{RnBi}} \; + \;\Updelta A_{\text{Bi}} $$

4 $$ A_{\text{mPo}} = \;A_{\text{RnPo}} \; + \;\Updelta A_{\text{Po}} $$

However, because of the relatively long half-live of ^210^Pb (22.3 years) in comparison to average aerosol residence times (several days), these parts of the total activities—Δ*A*_i_ of ^210^Pb, ^210^Bi and ^210^Po, should be constant when solid particles exist in the air. Therefore, the activity relations between these radionuclides, according to the one box model in the steady state, concern *A*_Rni_ parts only. Because of the above mentioned conditions, the activity of ^210^Pb—*A*_RnPb_ will also be constant, and for the remaining *A*_RnBi_ and *A*_RnPo_ activities, according to this model, one can write:

5 $$ A_{\text{Rn Pb}} \; = \;A_{\text{RnBi}} \;\left( {\frac{1}{{T_{\text{RC}} \lambda_{\text{Bi}} }} + 1} \right) $$

6 $$ A_{\text{RnBi}} = A_{\text{RnPo}} \left( {\frac{1}{{T_{\text{RC}} \lambda_{\text{Po}} }} + 1} \right) $$

where λ_Bi_ and λ_Po_ denote decay constants of ^210^Bi and ^210^Po, respectively, and T_RC_—residence time of aerosols, corrected for addition input of these radionuclides.

After introducing relations 2–4 to Eqs. 5 and 6 we obtain a pair of equations:

7 $$ A_{\text{mPb}} - X = (A_{\text{mBi}} - X)\left( {\frac{1}{{T_{\text{RC}} \lambda_{\text{Bi}} }} + 1} \right) $$

8 $$ A_{\text{mBi}} - X = \left( {A_{\text{mPo}} - X} \right)\left( {\frac{1}{{T_{\text{RC}} \lambda_{\text{Po}} }} + 1} \right) $$

where *A*_mPb_, *A*_mBi_ and *A*_mPo_, denote the measured activities of these three radionuclides in the air.

The solution of these equations gives following expression for so called “corrected residence time”—*T*_RC_, which is identical to those proposed by Turekian [4] as well as by Poet et al. [3]

9 $$ T_{\text{RC}} = \frac{{A_{\text{Bi}} - A_{\text{Po}} }}{{(A_{\text{Pb}} - A_{\text{Bi}} )\lambda_{\text{Bi}} - (A_{\text{Bi}} - A_{\text{Po}} )\lambda_{\text{Po}} }}. $$

The results of *T*_RC_ calculations for the dust samples collected in the summer 2010 and 2011 from the Eq. 9 as well as the calculations of the residence times from the “classical” model on the basis of the separate ^210^Bi/^210^Pb—*T*_RBi_ and ^210^Po/^210^Pb ratios—*T*_RPo_ are presented in the Table 1.

**Table 1:** The residence times of the aerosols collected in Poland (Lodz)

Sample code	^210^Bi/^210^Pb activity ratio	*T*_RBi_-calculated from (^210^Bi/^210^Pb) (days)	^210^Po/^210^Pb activity ratio	*T*_RPo_-calculated from (^210^Po/^210^Pb) (days)	*T*_RC_ (days)
1016	0.48	6.68	0.096	20.5	25.4
1017	0.14	1.18	0.020	7.9	1.0
1018	0.60	10.85	0.029	10.3	9.5
1019	0.62	11.80	0.002	1.9	11.7
1020	0.59	10.41	0.122	34.5	8.6
1120	0.55	8.84	0.081	23.6	7.85
1121	0.46	6.20	0.057	17.4	5.60
1122	0.66	14.23	0.036	12.2	13.9
1123	0.49	7.18	0.024	9.1	7.1
1124	0.40	4.83	0.029	10.4	4.6
1125	0.64	12.85	0.064	19.2	12.3
1126	0.68	14.99	0.060	18.1	14.7
1127	0.36	4.12	0.062	18.6	3.5
1128	0.35	3.84	0.058	17.7	3.25

It is evident that both ^210^Bi/^210^Pb and ^210^Po/^210^Pb methods give overestimated aerosol residence times when they are applied separately. However, the corrected resident times —T_RC_ are close to those calculated on the basis of ^210^Bi/^210^Pb ratios. The ^210^Bi concentrations in the surface air during the summer period (~0.3 mBq/m^3^) are approximately 5- times higher than those for ^210^Po (~0.06 mBq/m^3^). Therefore, the same additional activity input Δ*A* does not influence the *A*_Bi_/*A*_Pb_ ratio as much as it does for *A*_Po_/*A*_Pb_. The discrepancy of residence times between ^210^Bi/^210^Pb and ^210^Po/^210^Pb methods was attributed mainly to the additional sources of ^210^Po input to the atmosphere. It is worth mentioning that the ^210^Bi/^210^Pb method can be applied for aerosols with a residence time <30 days. The corrected residence times determined by us are consistent with the other literature data for aerosol residence times in the troposphere [7, 21].

### Calculation of the additional inflow of ^210^Pb, ^210^Bi and ^210^Po activities

After calculating *T*_RC_ values the additional activities *X* = Δ*A*_Pb_ = Δ*A*_Bi_ = Δ*A*_Po_ coming from other than ^222^Rn sources, can be evaluated from the Eqs. 8 or 9, by the following formula:

10 $$ X = A_{\text{Po}} - T_{\text{RC}} \lambda_{\text{Po}} \left[ {A_{\text{Bi}} - A_{\text{Po}} } \right] $$

where *A*_mPb, Bi_ is the measured activity of ^210^Pb and ^210^Bi, λ_Po_ is decay constant of ^210^Po.

The relative contribution [%] of this additional activity Δ*A*_i_/*A*_mi_ = Δ_i_ to the measured activities of ^210^Pb, ^210^Bi, and ^210^Po can be calculated from the following formulas:

11 $$ {\text{for}}^{ 210} {\text{Pb }}\quad \Updelta_{\text{Pb}} = \, X/A_{\text{mPb}} \; \times \; 100 $$

12 $$ {\text{for}}^{ 2 10} {\text{Bi}}\quad \Updelta_{\text{Bi}} \; = \;\left[ { 1- \, \lambda_{\text{Bi}} T_{\text{RC}} \left( {A_{\text{mPb}} /A_{\text{mBi}} - { 1}} \right)} \right]\; \times \; 100 $$

13 $$ {\text{and for}}^{ 2 10} {\text{Po}}\quad \, \Updelta_{\text{Po}} \; = \;\left[ { 1- \, \lambda_{\text{Po}} T_{\text{RC}} \left( {A_{\text{mBi}} /A_{\text{mPo}} - { 1}} \right)} \right]\; \times \; 100 $$

Such calculated values of *X* as well as its relative contribution to the total activities of these three radionuclides in the surface air are presented in Table 2.

**Table 2:** Additional ^210^Pb, ^210^Bi and ^210^Po activities and their relative contributions

No samples	*X* (μBq/m^3^)	Δ_Pb_ (%)	Δ_Bi_ (%)	Δ_Po_ (%)
1016	41.98	0.90	1.15	9.35
1017	1.57	1.94	13.85	97.0
1018	14.94	7.74	12.90	76.6
1019	18.51	0.85	1.36	20.1
1020	13.58	10.18	17.25	83.4
1120	23.7	6.26	11.38	77.3
1121	12.1	4.54	9.82	80.0
1122	7.7	1.83	2.76	30.5
1123	2.9	0.75	1.51	30.9
1124	7.2	2.08	5.20	71.0
1125	7.5	2.82	4.41	44.3
1126	3.5	1.42	2.11	23.9
1127	22.8	5.63	15.51	91.5
1128	19.0	5.34	15.42	91.9

As was expected, because of the low concentrations of ^210^Po in the lower troposphere, only in the case of this radionuclide does its additional inflow both from natural and anthropogenic sources play a substantial role (up to 97% of the total activity).

For continental surface air Moore et al. [9] estimated that the suspended soil particles could account for about the half of the additional sources of ^210^Po, whereas stratospheric injection and anthropogenic sources give together only ~10% contribution.

**Fig. 1:**
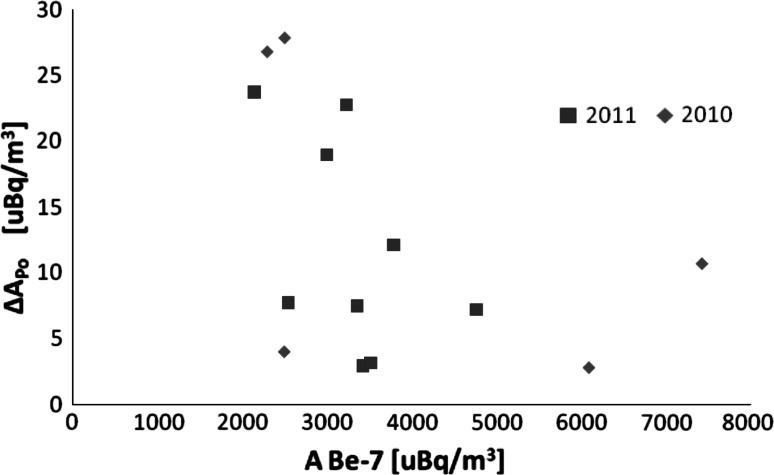
Values of the additional Δ*A*_Po_ activities are plotted against ^7^Be activities in Lodz aerosol samples

However, the relative importance of each of these sources (including plant decomposition) may be different for urban air. Apart from soil resuspension, the sources of the additional ^210^Po and ^210^Bi activities—*X*, in the case of Lodz can be emissions from the three coal fired power stations located within the city, as well as stratospheric injections. The stratospheric contribution can be appraised by checking the possible correlations between *X* = Δ*A*_Po_ and the surface air activity of the cosmogenic radionuclides ^7^Be or ^22^Na produced in the stratosphere–troposphere border. As is evident from Fig. 1, such a correlation does not exist.

On the other hand, the simultaneous comparative determinations of ^7^Be and ^22^Na in the surface aerosol samples can be used to trace the transport of the suspended solid particles from the stratosphere to the surface air. The number of the generated isotopes ^7^Be and ^22^Na depends on the intensity of cosmic radiation and also on the latitude, but their activity concentrations in the troposphere are strongly influenced by the intensity of precipitation. The seasonal changes in the activity ratio of ^7^Be/^22^Na in samples of aerosols allow us to calculate the retention time of these isotopes in the stratosphere—*T*_S_, if the residence times in the troposphere—*T*_RC_ are known. The ratio of their activity *R*_a_ in the surface air is given by the following formula [16, 17].

14 $$ R_{\text{a}} = \frac{{P_{\text{Be}} (1 - \exp ( - \lambda_{\text{Be}} T_{\text{S}} ))\exp ( - \lambda_{\text{Be}} T_{\text{RC}} )}}{{P_{\text{Na}} (1 - \exp ( - \lambda_{\text{Na}} T_{\text{S}} ))\exp ( - \lambda_{\text{Na}} T_{\text{RC}} )}} $$

where *P* theoretical nuclide production rate in the stratosphere, ^7^Be and ^22^Na respectively. The theoretical value of the *P*_Be_/*P*_Na_ is equal to 1.1 × 10^3^, *λ* is decay constants of ^7^Be and ^22^Na, respectively, *T*_RC_ is residence time in the atmosphere, *T*_S_ is residence time in the stratosphere.

The measured air activities of ^7^Be and ^22^Na as well as calculated values of *T*_S_ and total atmospheric residence times *T*_A_ = *T*_S_ + *T*_RC_ are summarized in the Table 3. The determined stratospheric residence times—*T*_S_ in the range 102–205 days are quite comparable with the scarce data for aerosol residence time in the stratosphere [15, 22].

**Table 3:** The stratospheric—*T*_S_ and total *T*_A_ atmospheric residence times of the aerosols transported from the stratosphere to the surface air

Sample code	Surface activity *A* (μBq/m^3^)		*R*_a_ ^7^Be/^22^Na activity ratio	Residence time (days)	
	^7^Be	^22^Na		*T*_S_	*T*_A_
1016	6,093	0.544	11,204	162.5	189.1
1017	7,429	0.543	13,674	205.3	206.3
1018	2,291	0.184	12,444	171.1	182.0
1019	2,496	0.269	9,286	172.8	184.8
1020	2,497	0.198	12,617	187.8	196.5
1120	2,139	0.476	4,493	102.6	110.4
1121	3,790	0.435	8,709	159.9	165.5
1122	2,541	0.248	10,234	173.8	187.7
1123	3,426	0.234	14,641	205.3	212.3
1124	4,768	0.928	5,136	111.7	116.3
1125	3,355	0.535	6,275	134.8	147.1
1126	3,515	0.270	13,019	196.7	211.8
1127	3,229	0.281	11,475	178.7	182.2
1128	2,999	0.240	12,496	182.9	186.2

The possible volcanic emissions of ^210^Po can be considered as point sources with irregular eruptive activity and supply to the stratosphere. The relatively long residence time of stratospheric aerosols enables its transport to non-seismic regions. However, during this time the activity of unsupported ^210^Po should decrease remarkably, and such influence on the total ^210^Po activity was observed only temporarily in regions adjacent to an active volcano [23, 24] and input of stratospheric ^210^Po to the lower tropospheric air in Central Poland is likely negligible. Therefore, the observed additional activities of ^210^Po in the urban air are likely caused by resuspension of surface soil and anthropogenic emissions mainly from coal-fired power plants. However, in order to quantify the relative contributions of these sources, additional measurements of so called “index trace elements” (specific for soil dust and coal combustion emissions) in aerosol samples should be done.

## Conclusions

The significant differences in the aerosol residence times calculated by ^210^Bi/^210^Pb and ^210^Po/^210^Pb methods can be explained by additional sources of ^210^Po in urban air to the ^222^Rn flux from soil. A simple modification of the one compartment model allows both the corrected troposphere aerosol residence times as well as the relative input of these sources to the total activity concentrations of ^210^Pb, ^210^Bi and ^210^Po radionuclides in the urban air to be calculated. Simultaneous measurement of two cosmogenic radionuclides, ^7^Be and ^22^Na, in aerosol samples also allows the stratospheric aerosol residence time to be also determined. The latter values can serve as a measure of the rate of exchange of air masses at the border of stratosphere-troposphere areas, which is important in the observation of long-term effects of particulate impurities on climate change.
